# Clinical and epidemiological aspects of SARS-CoV-2 infection among pregnant and postpartum women in Mozambique: a prospective cohort study

**DOI:** 10.1186/s12978-022-01469-9

**Published:** 2022-07-19

**Authors:** Charles M’poca Charles, Nafissa Bique Osman, Domingos Arijama, Benjamim Matingane, Tomás Sitoé, Darlene Kenga, Cesaltina Lorenzoni, Elvira Luís, Rodolfo de Carvalho Pacagnella, Jahit Sacarlal, for the Mozambique Study group of SARS-COV-2, Alfeu Passanduca, Alice Manjate, Aline Munezero, Cesaria Uassiquete, Filipe Majunta, Guilherme Moraes Nobrega, Ilza Cambaza, José Carlos, José Guilherme Cecatti, Maria Laura Costa, Renato Teixeira Souza, Sérgio Taúnde, Tufária Mussá

**Affiliations:** 1 Provincial Health Administration, DPS Manica, Chimoio, Manica Province Mozambique; 2 Department of Obstetrics and Gynecology, School of Medicine, University of Campinas, Campinas, São Paulo, Brazil; 3 Department of Obstetrics and Gynecology, Maputo Central Hospital, Maputo, Mozambique; 4 Department of Obstetrics and Gynecology, Faculty of Medicine, Eduardo Mondlane University, Maputo, Mozambique; 5 Department of Microbiology, Faculty of Medicine, Eduardo Mondlane University, Maputo, Mozambique; 6 Pathology Department, Faculty of Medicine, Eduardo Mondlane University, Maputo, Mozambique; 7 Pathological Anatomy Service, Maputo Central Hospital, Maputo, Mozambique

**Keywords:** COVID-19, Risk factors, Maternal and perinatal morbidity, Low-income country, Mozambique, COVID-19, Fatores de risco, Morbidade materna e perinatal, País de baixa renda, Moçambique, COVID-19, Factores de riesgo, Morbilidad materna y perinatal, País de bajos ingresos, Mozambique

## Abstract

**Background:**

Although there is a significant increase of evidence regarding the prevalence and impact of COVID-19 on maternal and perinatal outcomes, data on the effects of the pandemic on the obstetric population in sub-Saharan African countries are still scarce. Therefore, the study aims were to assess the prevalence and impact of COVID-19 on maternal and neonatal outcomes in the obstetric population at Central Hospital of Maputo (HCM), Mozambique.

**Methods:**

Prospective cohort study conducted at teaching and referral maternity, HCM, from 20 October 2020 to 22 July 2021. We collected maternal and perinatal outcomes up to 6 weeks postpartum of eligible women (pregnant and postpartum women—up to the 14th day postpartum) screened for COVID-19 (individual test for symptomatic participants and pool testing for asymptomatic). The primary outcome was maternal death, Severe Acute Respiratory Syndrome (SARS) and Intensive Care Unit (ICU) admission. We estimated the COVID-19 prevalence and the unadjusted RR (95% CI) for maternal and perinatal outcomes. We used the chi-square or Fisher's exact test to compare categorical variables (two-sided p-value < 0.05 for statistical significance).

**Results:**

We included 239 participants. The overall prevalence of COVID-19 was 9.2% (22/239) and in the symptomatic group was 32.4% (11/34). About 50% of the participants with COVID-19 were symptomatic. Moreover, the most frequent symptoms were dyspnoea (33.3%), cough (28.6%), anosmia (23.8%), and fever (19%). Not having a partner, being pregnant, and alcohol consumption were vulnerability factors for SARS-CoV-2 infection. The risk of adverse maternal and neonatal outcomes (abortion, foetal death, preterm birth, Apgar, and NICU admission) was not significantly increased with COVID-19. Moreover, we did not observe a significant difference in the primary outcomes (SARS, ICU admission and maternal death) between COVID-19 positive and COVID-19 negative groups.

**Conclusion:**

The prevalence of COVID-19 in the obstetric population is higher than in the general population, and fifty percent of pregnant and postpartum women with COVID-19 infection are asymptomatic. Not having a partner and alcohol consumption were factors of greatest vulnerability to SARS-COV-2 infection. Moreover, being pregnant versus postpartum was associated with increased vulnerability to COVID-19. Data suggest that pregnant women with COVID-19 may have a higher frequency of  COVID-19 infection, reinforcing the need for universal testing, adequate follow-up for this population, and increasing COVID-19 therapy facilities in Mozambique. Moreover, provide counselling during Antenatal care for COVID-19 preventive measures. However, more prospective and robust studies are needed to assess these findings.

## Plain language summary

The epidemiological pattern of the COVID-19 pandemic in Africa is heterogeneous, and many African countries are still struggling to establish efficient testing policy, guarantee sufficient laboratory supply and achieve or maintain adequate testing capacity. In addition, evidence suggests that sexual and reproductive health services were the most affected by the pandemic; this scenario might have devastating effects on maternal and perinatal health. Moreover, data from non-sub-Saharan countries the SARS-CoV-2 infection among pregnant and postpartum women is associated with an increased risk of adverse maternal and neonatal health (preterm birth, preeclampsia and maternal death).

Although there is a significant increase of evidence regarding the prevalence and impact of COVID-19 on maternal and perinatal health, data on the effects of this condition on the obstetric population in low-income countries are scarce. Therefore, the study objective were to assess the prevalence and impact of COVID-19 on maternal and neonatal health at referral maternity in Maputo, Mozambique.

Our findings suggest that the prevalence of COVID-19 in the obstetric population is higher than the general population, and most pregnant and postpartum women are asymptomatic. Being pregnant, not having a partner and alcohol consumption were factors of greatest vulnerability to SARS-COV-2 infection. Moreover, the risk of COVID-19 among pregnant was seven-fold higher than in postpartum women. Pregnant women with COVID-19 may have a higher frequency of adverse gestational outcomes (foetal death and abortion). Although the risk of adverse maternal outcomes (death, Severe Acute Respiratory Syndrome and Intensive Care Unit admission) did not differ significantly between the COVID-19 and COVID-19 negative groups, universal screening for COVID-19 should be implemented to ensure adequate management of pregnant women and newborns.

## Resumo

**Introdução:**

Apesar do aumento significativo de evidências relacionadas à prevalência e o impacto da COVID-19 nos desfechos maternos e perinatais, os dados dos efeitos da pandemia na população obstétrica dos países da África subsaariana ainda são escassos. Deste modo, o objetivo deste estudo é avaliar a prevalência e o impacto da COVID-19 nos desfechos maternos e perinatais da população obstétrica do Hospital Central de Maputo (HCM), Moçambique.

**Métodos:**

Realizamos um estudo de coorte prospectiva em uma maternidade de referência de 20 de outubro de 2020 a 22 de julho de 2021. Coletamos dados dos desfechos maternos e perinatais até a sexta semana pós-parto das mulheres elegíveis (gestantes e puérperas até ao décimo quarto dia do pós-parto) rastreadas para a COVID-19 (de forma individual para sintomáticas e ‘*pool testing*’ para assintomáticas). Os desfechos primários foram morte materna, síndrome respiratória aguda grave (SRAG) e admissão na unidade de tratamento intensivo (UTI). Estimamos a prevalência da COVID-19 e o risco relativo não ajustado (95% de IC) para os desfechos maternos e perinatais. Usamos o teste de qui-quadrado ou o teste exato de Fisher para comparar variáveis categóricas (consideramos p-valor bicaudal < 0.05 para a significância estatística).

**Resultados:**

Incluímos 239 participantes. A prevalência geral da COVID-19 foi de 9.2% (22/239) e no grupo das sintomáticas foi 32,4% (11/34). Cerca de 50% dos participantes com COVID-19 eram sintomáticos. Adicionalmente, os sintomas mais frequentes foram a dispneia (33.3%), tosse (28.6%), anosmia (23.8%), e febre (19%). Não ter parceiro, ser gestante, e consumir álcool, foram os fatores de vulnerabilidade à infeção pelo SARS-CoV-2. O risco para desfechos maternos e perinatais adversos (aborto, óbito fetal, parto prematuro, índice de Apgar baixo, e admissão na UTI neonatal) não foi significativamente acrescido pela COVID-19. Igualmente, não observamos diferenças significativas nos desfechos primários (SRAG, admissão na UTI e morte materna) entre o grupo COVID-19 positivo e o grupo COVID-19 negativo.

**Conclusão:**

A prevalência da COVID-19 na população obstétrica foi maior que na população geral, e cinquenta por cento das gestantes e mulheres no pós-parto com COVID-19 eram assintomáticas. Não ter parceiro e consumir álcool foram os fatores de maior vulnerabilidade à infeção pelo SARS-CoV-2. Adicionalmente, estar gestante comparado à puérpera foi associado ao maior risco da COVID-19. Os dados sugerem que mulheres gestantes com COVID-19 podem ter maior frequência de infecção por COVID-19, reforçando a necessidade de rastreio universal, seguimento adequado dessa população, e aumento de unidades de tratamento da COVID-19 em Moçambique. Igualmente, prover aconselhamento sobre as medidas preventivas da COVID-19 durante a consulta pré-natal. No entanto, são necessários mais estudos prospectivos e robustos para avaliar esses resultados.

## Resumen

**Introducción:**

A pesar del aumento significativo de evidencias relacionadas con la prevalencia y el impacto de la COVID-19 en los resultados maternos y perinatales, los datos sobre los efectos de la pandemia en la población obstétrica de los países del África Subsahariana aún son escasos. De este modo, el objetivo de este estudio es evaluar la prevalencia y el impacto del COVID-19 en los resultados maternos y perinatales en la población obstétrica del Hospital Central de Maputo (HCM), Mozambique.

**Métodos:**

Realizamos un estudio de cohorte prospectivo en una maternidad docente y de referencia, HCM, del 20 de octubre de 2020 al 22 de julio de 2021. Recolectamos datos sobre los resultados maternos y perinatales, hasta la sexta semana posparto, de mujeres elegibles (mujeres embarazadas y puérperas—hasta el decimocuarto día del posparto) examinadas para COVID-19 (de forma individual para sintomáticos y ‘*pool testing*’ para asintomáticos). Los resultados primarios fueron la muerte materna, el Síndrome Respiratorio Agudo Grave (SRAG) y el ingreso en la unidad de cuidados intensivos (UCI). Estimamos la prevalencia de COVID-19 y el riesgo relativo no ajustado (IC del 95%) para los resultados maternos y perinatales. Utilizamos la prueba de chi-cuadrado o la prueba exacta de Fisher para comparar variables categóricas (consideramos un valor de p de dos colas < 0.05 para la significación estadística).

**Resultados:**

Incluimos 239 participantes. La prevalencia global de COVID-19 fue del 9,2% (22/239) y en el grupo de sintomáticas fue del 32,4% (11/34). Alrededor del 50% de las participantes con COVID-19 eran sintomáticos. Además, los síntomas más frecuentes fueron disnea (33,3%), tos (28,6%), anosmia (23,8%) y fiebre (19%). No tener pareja, estar embarazada y consumir alcohol fueron los factores de vulnerabilidad a la infección por SARS-CoV-2. El riesgo de resultados maternos y perinatales adversos (aborto, muerte fetal, parto prematuro, bajo puntaje de Apgar e ingreso a la UCI neonatal) no aumentó significativamente por el COVID-19. Asimismo, no observamos diferencias significativas en los resultados primarios (SRAG, ingreso en UCI y muerte materna) entre el grupo COVID-19 positivo y el grupo COVID-19 negativo.

**Conclusión:**

La prevalencia de COVID-19 en la población obstétrica es mayor que en la población general, y el cincuenta por ciento de las gestantes y puérperas con COVID-19 se encontraban asintomáticas. No tener pareja y el consumo de alcohol fueron los factores de mayor vulnerabilidad a la infección por SARS-CoV-2. Además, estar embarazada en comparación con ser puérpera se asoció con un mayor riesgo de COVID-19. Los datos sugieren que las mujeres embarazadas con COVID-19 pueden tener una mayor frecuencia de infección, lo que refuerza la necesidad de detección universal, un seguimiento adecuado de esta población y un aumento de las unidades de tratamiento para el COVID-19 en Mozambique. Así como, brindar asesoría sobre las medidas preventivas del COVID-19 durante la consulta prenatal. Sin embargo, se necesitan más estudios prospectivos y robustos para evaluar estos resultados.

## Background

On the African continent, contrarily to previously developed prediction models, epidemiological data suggest that the progression of the first and second wave of the pandemic was slower, with fewer reported cases and lower disease-related mortality rate [1–3]. However, the epidemiological pattern of the COVID-19 pandemic in Africa is quite heterogeneous: about four-fifths (82.6%) of cases were reported in 9 of the 55 countries of the African Union (South Africa [38.3%], Morocco [15.9%], Tunisia [5.1%], Egypt [5.0%], Ethiopia [4.5%], Libya [3.6%], Algeria [3.6%], Kenya [3.5%], and Nigeria [3.2%]) [4]. This heterogeneous pattern is due to several factors as the low testing capacity, weak and inefficient epidemiological surveillance systems, and variation in the COVID-19 pandemic progression and response [4].

Many African countries are still struggling to establish efficient testing policy, guarantee sufficient laboratory supply and achieve or maintain the adequate testing capacity, testing at the level of ten negative tests to one positive (test per case ratio ≥ 10) [5, 6]. Nevertheless, during the pandemic, in most African countries, the COVID-19 diagnostic capacity was expanded through the GeneXpert platforms previously deployed to diagnose tuberculosis [7, 8], and Mozambique was not an exception.

In Mozambique, the first case of COVID-19 was reported on 22 March 2020. As of 19 September 2021, the Mozambique Ministry of Health (MoH) had reported 150,018 cases (tests per case ratio: 5.9) and 1903 COVID-19 deaths (case fatality ratio: 1.27%) [9]. Data suggest that sexual and reproductive health (SSR) services were the most affected by the pandemic, reducing or interrupting these services in more than 50% of cases [10]. Furthermore, this reduction in the provision of services might have devastating effects on maternal and perinatal health due to the increase in maternal and child mortality [11].

The prevalence of COVID-19 in pregnancy was estimated at 41% in symptom-based screening [12] or 7% in universal screening [13], finding from studies conducted in France and the United States of America, respectively. However, the COVID-19 prevalence can vary according to several factors, for example, epidemiological patterns of COVID-19 in the region and country, type of test used for SARS-CoV-2 detection and testing policy (universal or symptoms based screening), among others.

Data from two systematic reviews (SR) and meta-analysis suggested that the SARS-CoV-2 infection among pregnant and postpartum women is associated with an increased risk of adverse maternal and perinatal outcomes [14, 15]. In addition, data from Brazil and a living SR and meta-analysis suggested that pregnant women with advanced age, black race, obesity, and associated comorbidities such as hypertension, and diabetes mellitus have a higher risk of severity [15, 16]. These maternal and perinatal health effects are disproportionately higher in the low-income population, where health systems are fragile and less responsive to extreme adverse public health events [17]. For example, in Mozambique, hospitals are not adequately equipped (0.4% of hospitals with oxygen therapy available) and have low geographic accessibility [18].

Although there is a significant increase of evidence regarding the prevalence and impact of COVID-19 on maternal and perinatal outcomes [19], data on the effects of the pandemic on the obstetric population in sub-Saharan African countries are still scarce. Therefore, the present study aims to assess the prevalence and impact of COVID-19 on maternal and neonatal outcomes in the obstetric population admitted to the maternity hospital of the Central Hospital of Maputo (HCM), Mozambique.

## Methods

### Study population and study location

A prospective cohort study included pregnant and postpartum women (up to the 14th day of postpartum), asymptomatic or diagnosed with flu syndrome and/or suspected COVID-19, regardless of age, admitted to the Gynaecology and Obstetrics service of the Central Hospital of Maputo (HCM), Mozambique, from 20 October 2020 to 22 July 2021.

The HCM is a teaching and referral maternity hospital for the region and the country, with comprehensive obstetric care. The screening for SARS-CoV-19 infection in pregnant and postpartum women is similar to the general population, focused on symptomatic individuals or those with a history of contact with a positive case. We intentionally estimated a sample size of 300 participants (pairs of pregnant women and newborn) as the evidence on the effects of COVID-19 on pregnancy was paucity when we were implementing our study.

### Procedure

The study protocol included pregnant (regardless of the gestational age) and postpartum women (up to 14th day of puerperium) who attended the HCM obstetrical and gynaecological services and provided or signed the consent form. At hospital admission or soon after, the research team (nurses, resident doctors and consultant obstetrician) identified, invited and assessed for eligibility criteria all potentials participants (in the emergency room and/or patient wards) after giving complete study information, including procedures.

After reading and signing the informed consent form, the participants were asked to provide upper respiratory specimens for laboratory screening of SARS-CoV-2 infection. We excluded all women with invalid telephone numbers who did not accept providing upper respiratory specimens or withdrew their consent form during the study.

We collected nasopharyngeal and oropharyngeal specimens through swabs. For asymptomatic patients, we collected specimens in duplicate. After collecting the specimens, they were placed in a viral transport medium (VTM) containing antifungal and antibiotic supplements. We storage and shipped the specimens in cooler boxes on ice (at 2–8 °C) to the local laboratory for viral detection. All sample viral detection was done via GeneXpert platforms for COVID-19, and the results were available within 24 h (2 h for symptomatic participants and 24 h for asymptomatic participants).

The laboratory detention virus followed two approaches: the symptomatic participants and/or severe acute respiratory illness and high-risk contacts were individually tested. Conversely, samples from asymptomatic participants with no history of positive contact for COVID-19 were tested using a *pool testing* strategy. Pool testing is a technique in which specimens collected from different participants are organised into groups (‘pools’) and tested together [20]. At the time of study implementation, the data from the Mozambican obstetric population suggested that the prevalence of COVID-19 was around 6% [21]. Therefore, we estimated a pool of nine samples (P9S3) analysed in three stages [20, 22]. The pool tests positive was further divided into sub-pools of three specimens before retesting each specimen in the pool individually to determine which individual(s) are positive.

The specimens’ collection, processing, and testing were carried out by health professionals previously trained for this purpose and according to the standards recommended by the Ministry of Health Mozambique and the World Health Organization for collecting and handling clinical specimens for COVID-19 testing.

Subsequently, the included participants were allocated into two groups according to the test result. The COVID-19 positive group consisted of pregnant and postpartum women (up to the 14th day) with a positive test for SARS-CoV-2 infection. The second group (COVID-19 negative) consisted of pregnant and postpartum women with a negative test. During the follow-up, participants with a negative test could move to the COVID-19 positive group if they were positive for SARS-CoV-2 infection COVID-19 when retested.

During inclusion and follow-up (until the 6th week postpartum), data on sociodemographic and obstetric characteristics (including usual means of transportation, alcohol consumption, source of antenatal care, and underlying medical condition), clinical characteristics of SARS-CoV-2 infection, adverse maternal events, maternal and perinatal outcomes were collected by the research team. Data were collected through in person or /and telephone interview and medical record review. Moreover, all study data were collected and managed by the research team using REDCap (Research Electronic Data Capture) electronic data capture tools installed in smartphones (tablets) hosted at Eduardo Mondlane University, Maputo, Mozambique [23, 24].

The primary outcome was the severe maternal outcome (maternal death, SARS and UCI admission). Secondary outcomes were: pregnancy outcomes (abortion, foetal death), preterm birth, preeclampsia/ eclampsia, mode of delivery, Apgar, NICU admission, neonatal death, congenital anomaly and any composite of adverse pregnancy outcome (NICU admission, preterm birth, foetal death, neonatal death, miscarriage/abortion). In addition, we have considered potential confounders variables, other viral respiratory syndromes, history of adverse pregnancy outcomes, and all factors related to the three-delay model in obstetric care.

### Statistical analysis

We describe and compare the sociodemographic, obstetric and clinical characteristics of pregnant and postpartum women included in the study according to exposure (COVID-19 positive and COVID-19 negative groups). Likewise, we estimated the prevalence of COVID-19 in the general population, in the symptomatic and asymptomatic groups, and compared the clinical and severity characteristics in the group of symptomatic women according to the exposure group and estimated the level of significance (we considered Two-sided p-value < 0.05 as statistically significant).

We additionally have considered the time of symptom onset before admission, the duration of symptoms, the most prevalent symptoms, the type of management at the time of admission, admission to the intensive care unit and the presence of the severe acute respiratory syndrome. Finally, we estimated the unadjusted relative risk with a 95% interval to evaluate the risk of adverse maternal and perinatal outcomes. For comparisons of categorical variables, we used the Chi-square test or Fisher’s exact test when indicated. Statistical analyses were performed using the IBM SPSS statistic program (version 27.0).

### Ethical issues

The study protocol was approved by the Mozambique National Review Board (Letter of approval number 61/CNBS/2020). Moreover, all participants were fully informed regarding the study procedure and provided written or oral consent before their inclusion in the study. In addition, all participants had adequate clinical management (for SARS-CoV-2 positive cases) and psychological support when needed.

## Results

**Fig. 1:**
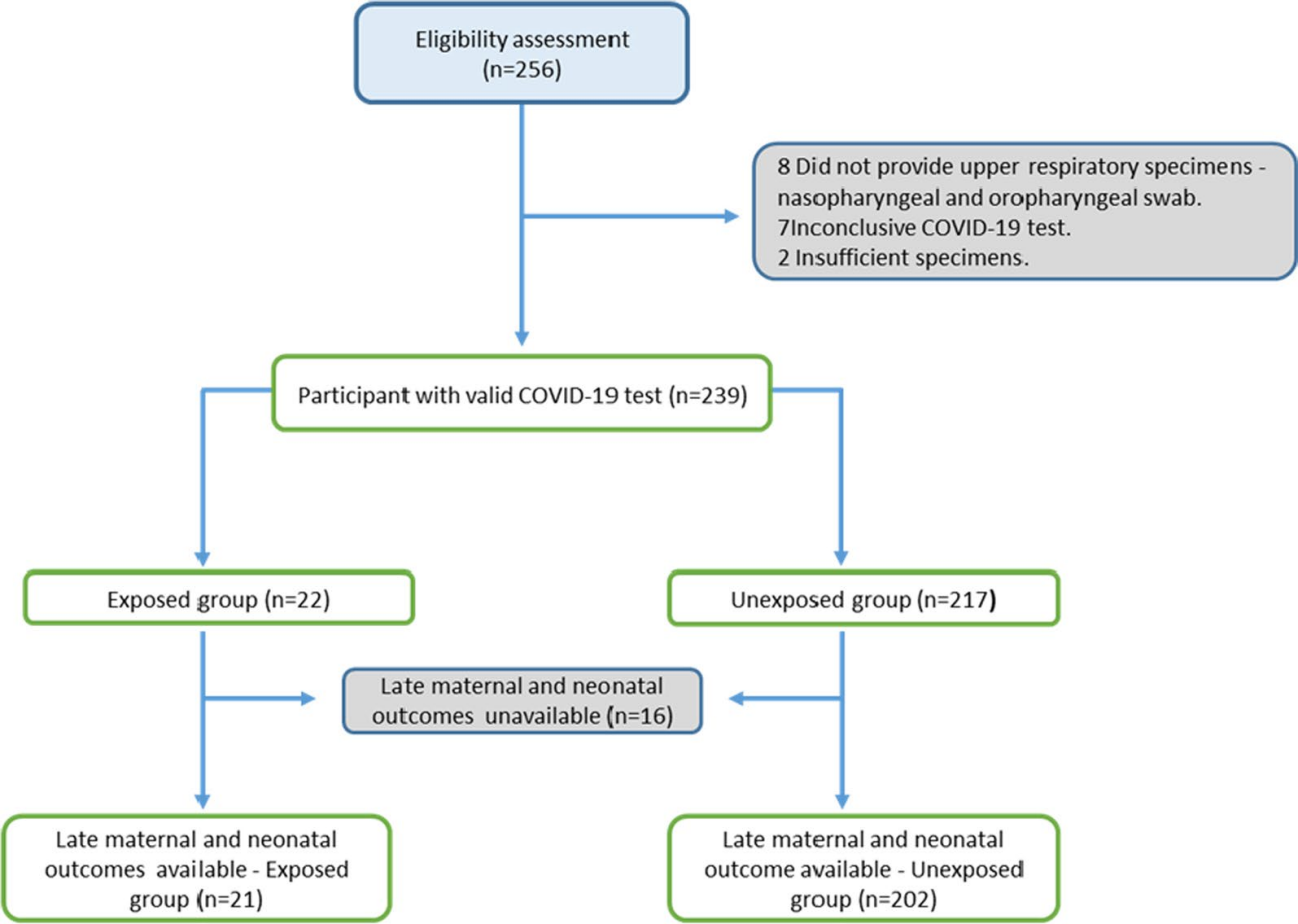
Study flowchart

We included 239 participants; 22 were COVID-19 positive, and 217 were COVID-19 negative. Maternal and neonatal outcomes were available in 93% of the included participants (223/239) (Fig. 1). The average age was 28 years (SD 6.1), and the majority of the population was Black (92.1% [220/239]).

At the time of study admission, about 37% (83/226) were pregnant, two-thirds of the participants had had at least four antenatal care consultations, and the majority (69.4% [150/216]) of the participants had prenatal consultations in public services (Table 1).

**Table 1:** Sociodemographic and obstetric characteristics of Maputo Central Hospital included in the study (n = 239)

Characteristics	Confirmed COVID-19 n = 22	Negative COVID-19 n = 217	*p*-value
Age^*a*^			
≤ 19	2 (9.1%)	15 (7.4%)	0.326
20–35	14 (63.6%)	155 (76.0%)	
> 35	6 (27.3%)	34 (16.7%)	
Ethnicity^b^			
Black	20 (95.2%)	200 (98.0%)	0.390
Non-black	1 (4.8%)	4 (2.0%)	
Area of residence^c^			
Peri-urban	19 (86.4%)	183 (90.6%)	0.461
Urban	3 (13.6%)	19(9.4%)	
Marital status^d^			
With partner	13 (61.9%)	164 (80.8%)	**0.043**
Without partner	8 (38.1%)	39 (19.2%)	
Schooling^e^			
None or primary or secondary	12 (63.2%)	131 (64.9%)	0.883
College or more	7 (36.8%)	71 (35.1%)	
Usual means of transport^f^			
Public	10 (52.6%)	140 (71.1%)	0.096
Private	9 (47.4%)	57 (28.9%)	
Source of antenatal care^g^			
Public	17 (68.9%)	181 (94.3%)	0.112
Private	3 (15.0%)	11 (5.7%)	
ANC consultation^h^			
None	1 (5.0%)	7 (3.5%)	0.667
1–3	5 (25.0%)	60 (30.0%)	
≥ 4	14 (70.0%)	133 (66.5%)	
Provenience^i^			
Home	18 (81.8%)	168 (82.8%)	1.00
Referral	4 (18.2%)	35 (17.2%)	
Parity^j^			
0	12 (57.1%)	87(42.0%)	0.447
1–2	7 (33.3%)	98 (47.3%)	
≥ 3	2 (9.5%)	22 (10.6%)	
Planned pregnancy^k^	11 (52.4%)	134 (65.4%)	0.237
Multiple pregnancy	1 (4.8%)	7 (3.4%)	0.544
Pregnancy status at enrolment^l^			
Pregnancy	17 (81.0%)	66 (32.2%)	**0.000**
Post-partum	4 (19.0%)	139 (67.8%)	
Chronic hypertension	1 (4.5%)	6 (2.8%)	0.496
Pre-existing diabetes	0 (0.0%)	0 (0.0%)	NA
Asthma	1 (4.5%)	2 (0.9%)	0.252
Anemia	0 (0.0%)	10 (4.6%)	0.427
HIV	2 (9.1%)	28 (12.9%)	0.749
Alcohol drinking^n^	13 (65.0%)	62 (31.0%)	**0.002**
Symptoms^m^			
Yes	11 (52.4%)	22 (10.9%)	**0.000**
No	10 (47.6%)	180 (89.1)	

Missing information: ^a^13, ^b^14, ^c^15, ^d^15, ^e^18, ^f^23, ^g^27, ^h^19, ^i^19, ^j^13, ^k^13, ^l^13, ^m^1, ^n^19

**Fig. 2:**
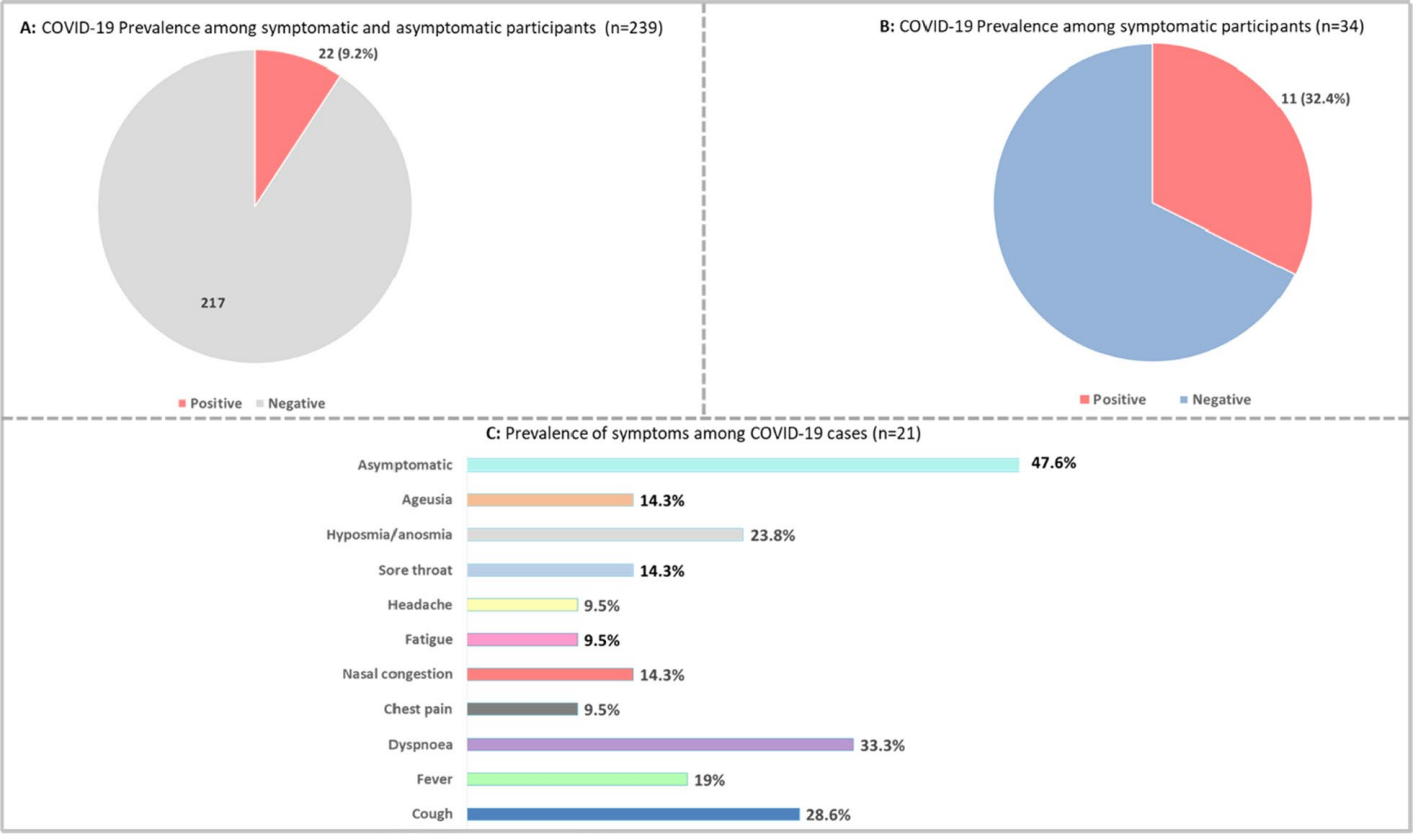
Prevalence of COVID-19 and symptoms among pregnant and postpartum women

The overall prevalence of COVID-19 was 9.2% (22/239) and in the symptomatic group was 32.4% (11/34) (Fig. 2A and B). About 48% of the participants with COVID-19 were asymptomatic (Fig. 2C). Dyspnoea (33.3%), cough (28.6%), anosmia (23.8%), and fever (19%) were more frequent symptoms. Hyposmia/anosmia (p-value = 0.00) and ageusia (p-value = 0.02) were symptoms statistically associated with COVID-19 diagnoses (Table 2). We were unable to assess maternal and perinatal outcome for 16 participants.

**Table 2:** Clinical features and severity of COVID-19 infection among symptomatic women (n = 32)

Clinical features and severity	Confirmed COVID-19 n = 10	Negative COVID-19 n = 22	*p*-value#
Number of days with symptoms before enrolment			
< 7	7 (70.0%)	14 (63.6%)	1.00
≥ 7	3(30.0%)	8 (36.4%)	
Symptoms (Prevalence ≥ 20%)			
Cough	6 (60%)	13 (63.6%)	1.00
Fever	4(40%)	10 (45.5%)	1.00
Dyspnoea	7 (70%)	7 (31.8%)	0.06
Chest pain	2 (20%)	2 (9.1%)	0.57
Nasal congestion	3 (30%)	5 (22.7%)	1.00
Fatigue	2 (20%)	2 (9.1%)	0.57
Headache	2 (20%)	5 (22.7%)	1.00
Sore throat	3 (30%)	2 (9.1%)	0.29
Hyposmia /anosmia	5 (50%)	0 (0%)	**0.00**
Ageusia	3 (30%)	0 (0%)	**0.02**
Initial management			
Discharge from ER	4 (40.0%)	1 (4.5%)	0.087
Ward admission or Labour ward	5 (50.0%)	17 (77.3%)	
ICU admission	1 (10.0%)	4 (18.2%)	
SARS	1 (10.5%)	3 (14.3%)	1.00
ICU admission at any time	1 (10.0%)	7 (31.8%)	0.38

^#^Fisher’s exact test

The sociodemographic factors significantly associated with increased risk of SARC-CoV-2 infection were not having a partner, being pregnant, and consuming alcohol during pregnancy (Table 1). Moreover, the risk of COVID-19 among pregnant was seven-fold higher than in postpartum women (RR: 7.32 [2.54–21.03] 95% CI, p-value = 0.0002).There were non-significant differences between the COVID-19 positive and COVID-19 negative groups for the following outcomes: duration of symptoms, initial management, the presence of severe acute respiratory syndrome and admission to the intensive care unit at any time (Table 2). The risk of adverse maternal and neonatal outcomes (abortion, foetal death, preterm birth, apgar, and NICU admission) was not significantly increased with COVID-19 (Table 3). Moreover, We found no s significant difference between COVID-19 positive and COVID-19 negative groups for the remain maternal and perinatal outcomes (Table 3). Moreover, during the cohort follow-up, we did not record any cases of maternal death.

**Table 3:** Risk estimates for adverse pregnancy and neonatal outcomes according to COVID-19 exposure (n = 223)

Pregnancy outcomes	COVID-19 positive n = 21	COVID-19 negative n = 202	p-value
Pregnancy outcome			
Abortion	1 (4.8%)	0 (0.0%)	0.08
Foetal Death	2 (9.5%)	4 (2.0%)	0.09
Live birth	18 (85.7%)	198 (98.0%)	Ref.
Preterm birth^a^	3 (15.0%)	49 (25.4%)	0.42
Pre-eclampsia	1 (4.8%)	52 (25.7%)	0.03
Mode of delivery			
Vaginal birth	18 (85.7%)	154 (76.2%)	Ref.
Elective C-section	2 (9.5%)	21 (10.4%)	1
Intrapartum C-section	1 (4.8%)	27 (13.4%)	0.48
Apgar < 7 at 1st minute^b^	1 (5.9%)	36 (19.1%)	0.31
Apgar < 7 at 5th minute^b^	0 (0.0%)	14 (7.4%)	–
Neonatal respiratory distress^c^	0 (0.0%)	25 (14.7%)	–
Neonatal mechanical ventilation^d^	0 (0.0%)	8 (4.7%)	–
NICU admission^e^	2 (12.5%)	36 (20.6%)	0.74
Any neonatal morbidity	2 (9.5%)	43 (21.3%)	0.26
Congenital anomaly	0 (0.0%)	2 (1.12%)	–
Neonatal death	0 (0.0%)	5 (2.9%)	–
Any APO/WHO*	7 (33.3%)	67 (33.2%)	0.98
Any gestational intercurrence**	5 (23.8%)	48 (23.8%)	0.99

Missing information ^a^10, ^b^18, ^c^37, ^d^35, ^e^25

*APO (any adverse pregnancy outcome): NICU admission, preterm birth, foetal death, neonatal death, miscarriage/abortion

**Hyperemesis, Foetal growth restriction, haemorrhage during pregnancy, threatened preterm labour

## Discussion

This prospective and exploratory study report the prevalence of COVID-19 in pregnancy and its impact on maternal and perinatal health in the obstetric population of Maputo, Mozambique. The overall prevalence of COVID-19 in pregnant and postpartum women was 9.2%. Almost half of the population was asymptomatic at the time of diagnosis. In addition, the sociodemographic and gestational factors commonly associated with greater vulnerability to SARS-CoV-2 infection were being pregnant, alcohol consumption, and not having a partner.

These data suggest that the overall prevalence of COVID-19 in pregnant and postpartum women is higher than the general Mozambican population, which was 2–4% [25]. Likewise, this prevalence is relatively higher than that of the study in pregnant and postpartum women, also carried out in Maputo city [21]. The difference in the COVID-19 prevalence might be due to the testing strategy, as the studies previously conducted in Mozambique (in general and obstetric population) were seroepidemiologic, and the COVID-19 pandemic magnitude in the country at the time of the studies implementation.

Conversely, our findings are similar to the results of the systematic review by Allotey and colleagues and another epidemiological study carried out in Zambia, which estimated an overall prevalence of COVID-19 in pregnant and postpartum women of 10% and 11.7%, respectively [15, 26].

The prevalence of COVID-19 was 32.4% in the group of symptomatic women at study admission. These findings are similar to other studies in which testing was based on clinical symptoms [15, 27]. Therefore, these data reinforce that the best testing approach is universal in places where resources are available to ensure proper management of pregnant women and newborns once even asymptomatic patients have an increased risk of maternal outcomes, maternal morbidity (RR: 1.24 [1.00–1.54] 95% CI) and preeclampsia (RR: 1.63 [1.01–2.63] 95% CI) [28].

Our data show that the risk of adverse maternal and neonatal outcomes (abortion, foetal death, preterm birth, Apgar, and NICU admission) was not significantly increased with COVID-19. However, our finding suggests a higher frequency of foetal death (9.5% vs 2.0%) and abortion (4.8% vs 0%) in the COVID-19 positive group. These findings are similar to the systematic review, which estimated increased risk of stillbirth (OR: 1.29 [1·06–1·58] 95% CI) [14] and (RR 2.84 [1.25–6.45]) [15]. The higher frequency of adverse maternal outcomes observed in our cohort may be due to the third delay (receiving adequate and appropriate treatment) [29], which the COVID-19 pandemic has exacerbated.

We did not observe significant differences in the risk of admission to the intensive care unit, development of severe acute respiratory syndrome, preeclampsia, preterm birth, NICU admission and neonatal death between the COVID-19 positive and COVID-19 negative groups. Our data are similar to systematic reviews [14, 30] and individual studies [28]. On the other hand, our findings differ from those of other published studies for maternal ICU admission outcomes, preeclampsia, which increased risk in pregnant women with COVID-19 [30, 31].

The major limitation of this study is related to the sample size. The sample size was small as it might not have the power to detect a difference between the exposure and non-exposure groups for some maternal and perinatal outcomes. Furthermore, although we have estimated a sample size of 300 participants (pairs of pregnant women and newborn), a scarcity of laboratory supplies (SARS-CoV-2 GeneXpert cartridges) at the national level hindered the study implementation. Therefore, reinforcing the difficulty of implementing prospective studies in places with few resources. In addition, the scarcity of SARS-CoV-2 GeneXpert cartridges might have influenced the lower test per COVID-19 case ratio de 5.8 observed in Mozambique, which is almost half of the recommended ratio. The second limitation would be related to the testing strategy for the asymptomatic participant. Although the pooling test strategy might raise some concerns regarding the test performance [32], studies suggest that this testing modality could be implemented without compromising the sensitivity and specificity of the test [20, 33, 34]. We consider that this technique should be implemented in a low-resource setting (for example, Mozambique) to upscale the test capacity. Moreover, we implemented the study in a referral hospital with comprehensive and specialised obstetric care. In addition, we included a population mainly from the urban region; thus, the sample might not represent the entire population. Therefore, the study finding should be interpreted with caution, limiting their generalizability.

Conversely, our study has some strengths. First, we conducted a prospective study. Prospectively collected data were used to implement an adequate measure and appropriate COVID-19 cases management at the hospital level, with early isolation of positive cases, rational use of protective equipment and reduction of COVID-19 hospital transmission. Second, to the best of our knowledge, this is the first study developed in low-resource countries in sub-Saharan Africa and might be used as a baseline for future studies. Third, our study highlighted the role of modifiable factors (alcohol consumption) and the risk of SARS-CoV-2 infection. Likewise, the evidence of a risk increase of COVID-19 among pregnant women can raise awareness for greater attention to this group of patients and guide the construction and implementation of public policies to deal with COVID-19 in the obstetric population at the local and regional level.

## Conclusions

The prevalence of COVID-19 in the obstetric population is higher than in the general population, and fifty percent of pregnant and postpartum women with COVID-19 infection are asymptomatic. Not having a partner and alcohol consumption were factors of greatest vulnerability to SARS-COV-2 infection. Moreover, being pregnant versus postpartum was associated with increased vulnerability to COVID-19. Data suggest that pregnant women with COVID-19 may have a higher frequency of  COVID-19 infection, reinforcing the need for universal testing, adequate follow-up for this population, and increasing COVID-19 therapy facilities in Mozambique. Moreover, provide counselling during Antenatal care for COVID-19. However, more prospective and robust studies are needed to assess these findings.

## Data Availability

The datasets used during the current study are available from the corresponding author on reasonable request.

## Abbreviations

ICF: Informed Consent Form
HCM: Maputo Central Hospital
MoH: Mozambique Ministry of Health
NICU: Neonatal Intensive Care Unit
P9S3: Pool of nine samples analysed in three stages
REDCap: Research Electronic Data Capture
SRH: Sexual and reproductive health
VTM: Viral transport medium
